# Psychometric evaluation of the Chinese version of the stressors in breast cancer scale: a translation and validation study

**DOI:** 10.1186/s12889-024-18000-3

**Published:** 2024-02-09

**Authors:** Wenqi Hu, Jiahui Bao, Xiaolin Yang, Mao Ye

**Affiliations:** 1 The First Affiliated Hospital of China Medical University, Liaoning, China; 2 China Medical University, Liaoning, China

**Keywords:** Breast cancer, Translation, Cross-cultural adaptation, Psychometric assessment, Stressors

## Abstract

**Objective:**

To translate the Stressors in Breast Cancer Scale (SBCS) from English to Chinese and assess its psychometric properties.

**Methods:**

The Brislin’s translation model was applied to perform forward translation, back translation, cross-cultural adaptation, Whereas the Chinese version of the SBCS was formed by conducting pre-testing. A cohort of 878 breast cancer patients participated in this methodological study. Content validity, construct validity, convergent validity, discriminant validity, and criterion-related validity were used to establish validity. Internal consistency reliability, split-half reliability, and test-retest reliability were used to establish reliability.

**Results:**

The final scale contained five dimensions and 24 items, including interpersonal relationship and healthcare strains, worries and concerns about the future, physical appearance and sex strains, daily difficulties and health. The average content validity index of the scale was 0.975. The goodness-of-fit index (χ2/DF = 2.416, RMSEA = 0.057, GFI = 0.896, CFI = 0.947, IFI = 0.947, and TLI = 0.939) indicated that the model was well-fitted. The composite reliability (CR) of the dimensions ranged from 0.825 to 0.934, the average variance extracted (AVE) ranged from 0.539 to 0.712, and the correlation coefficients of each dimension with the other dimensions were less than the square root of the AVE for that dimension. The Criterion-related validity was 0.511. The Cronbach’s alpha was 0.938, and the dimensions ranged from 0.779 to 0.900. Split-half reliability was 0.853, with dimensions ranging from 0.761 to 0.892. Test-retest reliability was 0.855.

**Conclusions:**

The Chinese version of the SBCS has good reliability and validity, which can be applied to the assessment of stressors in breast cancer patients in China.

## Introduction

Breast cancer is the second leading cause of cancer-related deaths in women. A survey by the World Health Organization (WHO) shows that breast cancer accounts for 11.7% of all new cancer patients, bringing a heavy disease burden to patients [1]. In China, breast cancer ranks fifth in the incidence of malignant tumors and first in the incidence of female malignant tumors [2, 3]. At present, the incidence of female breast cancer in China is on the rise, and the overall situation is not optimistic. With the progress of breast cancer screening methods and treatment measures, the five-year survival rate of patients is as high as 68.1% ~ 93.2% [4]. However, the fear of disease, postoperative body image disorders, toxic side effects of treatment, economic burden, uncertainty of recovery and other factors lead to certain psychological pressure on patients [5–9].

Stress is defined both as a response and a stimulus [10, 11], and stressors are factors causing stress responses, such as life events, chronic strain, and daily hassles [12, 13]. Alagizy et al. [14] found that 78.1% of breast cancer patients were at moderate or high levels of stress. Abdollahi et al. [15] evaluated 210 breast cancer patients who underwent biopsy or mastectomy, which showed that the patients had high levels of stress. Gao et al. [16] investigated 407 breast cancer patients and found that only 1.2% of patients were non-stressed or mildly stressed. For breast cancer patients, the level of stress not only affects disease regression and quality of life [17, 18], but also increases the risk of cancer recurrence and shortens survival [19, 20]. Therefore, it is essential to timely and accurately assess the stress level of breast cancer patients, which can provide an effective reference for early development of targeted interventions.

Stress is often assessed by self-report measures, and researchers have developed several stress assessment tools specific to the cancer context, such as the Questionnaire for Stress in Cancer Patients (QSC-R23) [21], the Newly Diagnosed Breast Cancer Symptoms Stress Scale (NDBCSS) [22], and the Stressors in Breast Cancer Scale (SBCS) [23].The QSC-R23 is a 23-item scale for assessing cancer-related daily stressors, and its psychometric properties have been examined in different cancer populations, such as breast, hematological oncology, gynecological, thyroid, and urological cancers [24, 25]. However, its use is limited to German-speaking patients because to our knowledge, no psychometric information exists on its translation into English [26]. In addition, it is applicable to all cancer patients, does not address the specifics of breast cancer (e.g., breast-related appearance), and is inadequately adapted to the specificities of these patients. The NDBCSS is a 17-item scale for assessing stress perceptions in women newly diagnosed, later translated into Greek [27]. As its name indicates, it focuses only on newly diagnosed breast cancer patients and does not explore the stress that individuals may experience at different stages. Whereas, the SBCS, which was developed specifically for breast cancer patients, considers the specificities of breast cancer patients such as problems related to breast appearance and women’s interest in sex. Furthermore, the SBCS is applicable to the entire course of breast cancer, including survival after successful treatment.

At present, there is still a lack of specific tools for stress assessment of breast cancer patients in China, and general scales such as the Perceived Stress Scale (PSS) and the stress subscale of the Depression and Anxiety Stress Scale (DASS-21) are mostly used. The PSS is the most popular tool for assessing perceived stress. It has been translated into various languages such as German, Japanese, Chinese, Thai, Spanish, etc., and has been widely used in different populations in different countries [28]. The DASS-21, to our knowledge, has also been translated into more than fifty non-English languages, and has obtained extensive usage around the world [29]. Both of them are widely used in breast cancer patients [28, 30–32]. However, they were developed for the general population, and for breast cancer patients they may not be able to accurately and effectively measure the stress level of this specific population on their own, and should be complemented by other more specific ones.

Therefore, this study aimed to translate the English version of SBCS into Chinese and further assess its psychometric properties, in order to more systematically assess the stress status of breast cancer patients in China and to provide rational evidence for the development of effective interventions, thus helping patients to relieve stress and improve quality of life.

## Methods

### Study design and participants

This study aimed to translate the SBCS scale and test its reliability and validity. This methodological study used convenience sampling to select breast cancer patients hospitalized in the breast surgery, oncology, and radiotherapy departments of three teaching hospitals in Shenyang City from July 2023 to August 2023.The inclusion criteria were as follows: (1) Age ≥ 18 years; (2) The pathological diagnosis was confirmed as breast cancer; (3) No cognitive or psychological impairment; (4) Informed consent. Exclusion criteria were as follows: (1) The answer time < 2 min; (2) Incomplete completion of the questionnaire. The sample size was established in accordance with the general rules of the factor analysis procedure, which required a minimum of 10 respondents to be recruited for each project, although larger samples were desirable [33]. Finally, 878 participants were recruited.

### Translation and cross-cultural adaptation

This study has been authorized by the original authors to translate the English version of SBCS in two stages following Brislin’s translation model [34]. In the first stage, the English version of SBCS was independently translated into Chinese by two native Chinese-speaking medical doctors who were proficient in English (the two translators were clinical PhDs who had passed the CET-6 and had worked in the breast surgical department for more than 5 years). Then, the first author (a nursing graduate student who had passed the CET-6) and the two translators compared the two versions of the initial translation, discussed the differences and problems with the initial translators, and synthesized them into one forward translation. In the second stage, the synthesized forward-translated version was independently back-translated by two bilingual medical oncologists who had not been exposed to the original version (the two back-translators were both PhD oncologists and had been working in the United States for more than 10 years). The first author and the two translators then compared and synthesized the two versions of the back-translation. Finally, a panel of experts (a nursing professor, two associate chief nurses, two nurse practitioners charged with clinical work for more than 10 years, all with postgraduate degrees or above) compared and evaluated the synthesized back-translated version with the original version in order to achieve linguistic and conceptual equivalence between the two versions. During the translation procedure, the expert panel agreed that the SBCS item descriptions matched the Chinese language very well. Therefore, no items were modified for cross-cultural adaptation.

### Pre-testing

We conveniently recruited 30 breast cancer patients for pre-testing. Each patient completed the paper questionnaire independently or with the help of the researcher. The patients were interviewed during the completion process, which included the scale guidelines, the comprehensibility, readability, and fluency of the items and options, as well as the modification suggestions for the scale. All patients stated that the items were easy to understand and that there were no suggestions for modification, so no modifications were required for the scale.

## Measures

### General information questionnaire

A self-designed general information questionnaire was created based on the review of relevant literature and expert discussion combined with clinical practice. It consists of two parts: (1) Sociodemographic information: age, ethnicity, education, marital status, religious beliefs, primary caregiver, health insurance and occupation; (2) clinical information: breast cancer stage, time since diagnosis, recurrence, current treatment methods and surgical methods.

### Perceived stress scale (PSS)

Cohen developed this scale in 1983 to assess an individual’s level of stress in response to a stressful event [35]. This study used the Chinese version of the Perceived Stress Scale, which consists of 2 dimensions of tension (7 items) and loss of control (7 items) [36]. The 14-item scale is based on a 5-point Likert-type scale from 0 (never) to 4 (always), with a total score of 0 to 56 points. A higher scale total score indicates higher levels of perceived stress. A total score of 0–28 indicates a normal level of perceived stress, a total score of 29–42 indicates a high level of perceived stress, and a total score of 43–56 indicates a high level of perceived stress. Cronbach′s alpha coefficient for the original scale was 0.78, and in this study, it was 0.71.

### Stressors in breast Cancer scale (SBCS)

Cerezo developed this scale in 2023 to assess stressors in breast cancer patients [23]. The scale consists of 24 items in 5 dimensions (physical appearance and sex strains, health and daily difficulties, interpersonal relationship strains, healthcare strains, and worries and concerns about the future). The items were rated on a 5-point Likert-type scale (1 = not at all stressful or irrelevant to me; 5 = very stressful). Higher scale scores indicate higher stress. The overall Cronbach’s alpha coefficient for the original scale was 0.95, with dimensions ranging from 0.83 to 0.89, and in this study, it was 0.938 and 0.779 ~ 0.900, respectively.

### Data collection

Researchers explained the purpose of the study to the patients, and the paper questionnaires were distributed only after obtaining consent. All questionnaires were distributed and collected on the spot, and researchers used unified guidelines to explain the questionnaire items and filling methods. After completing the questionnaire, collect and organize them in time, and eliminate invalid questionnaires. It took about 10–15 min to complete the questionnaire. This study was approved by the Ethics Committee of the First Affiliated Hospital of China Medical University (approval number: [2023] No.391).

### Statistical analysis

EpiData 3.1 was used for data entry, and SPSS 26.0 (IBM Corp, Armonk, NY) and AMOS 24.0(IBM Corporation)software were used for statistical analysis. Descriptive statistics were used to assess the characteristics of the participants. *P* < 0.05 was statistically significant.

### Item analysis

Critical ratio method (CR): reflecting the discriminatory degree of the scale items, the total scores of the Chinese version of the SBCS scale were ranked from low to high, and the first 27% (< 37 points) of the total scores were regarded as the subgroups of low, and the last 27% (> 59 points) were regarded as the subgroups of high, and an independent samples t-test was carried out. If the CR value was > 3 and *P* < 0.05, the item was retained [33, 37]; correlation coefficient method: Pearson correlation analysis was used to calculate the correlation coefficient between the scores of each item and the total scores of the scale, r. If r was > 0.4 and *P* < 0.05, the item was retained [33]; homogeneity test (Cronbach’s alpha coefficient method): if the Cronbach’s alpha coefficient of the scale increased by deleting an item, the item was excluded [33].

### Validity analysis

Content validity: Five experts in different fields were invited to conduct 2 rounds of expert consultation on the Chinese version of the SBCS. Two of them were experts in clinical medicine, one in nursing, one in psychology, and one in public health, and all experts had worked in their respective fields for 5 years or more. Content validity was scored on a 4-point scale: not relevant = 1 point, weakly relevant = 2 points, more relevant = 3 points, and very relevant = 4 points. Each expert evaluated the items, the number of experts who gave a score of 3 or 4 divided by the total number of experts was the item-level Content Validity Index (I-CVI); the Scale-Level Content Validity Index/Average (S-CVI/Ave) was the mean of the I-CVI over all items. I-CVI ≥ 0.78 [38] and S-CVI/Ave ≥ 0.90 [39] indicated that the content validity of the scale was good.

Construct validity: 878 patients were randomized into two groups, exploratory factor analysis (EFA = 439) and confirmatory factor analysis (CFA = 439). EFA was used to evaluate the construct validity of the Chinese version of SBCS. Sampling adequacy was examined using the Kaiser-Meyer-Olkin (KMO) and the Bartlett’s test of sphericity prior to EFA analysis. When the Bartlett’s test of sphericity was significant (*P* < 0.05) and the KMO value was greater than 0.60 [40], Principal Component Analysis (PCA) was performed using maximum variance orthogonal rotation in order to extract factors with factor loadings greater than 0.40 and eigenvalues greater than 1.0 [41]. Validation factor analysis was used for the evaluation of model fitness.

Convergent validity and discriminant validity: If the average variance extracted (AVE)>0.5,composite reliability (CR) value>0.6, it indicated good convergent validity [42]; if the$\sqrt{AVE}$of a variable was greater than the correlation coefficient between the variable and all other variables, it indicated good discriminant validity [42].

Criterion-related validity: the correlation coefficient r was used to indicate the degree of correlation between the Chinese version of SBCS and the Chinese version of PSS. Criterion-related validity between 0.4 and 0.7 was desirable [43].

### Reliability analysis

Internal consistency reliability was evaluated using Cronbach’s alpha coefficient and split-half reliability, split-half reliability was evaluated using the correlation coefficient of the total score of odd-even items, and test-retest reliability was evaluated using the intraclass correlation coefficient (ICC). Cronbach’s alpha coefficient > 0.70 [44–46] and ICC > 0.80 were considered acceptable [47]. In this study, 30 breast cancer patients were retested at 2-week intervals, and Pearson correlation was used to analyze the correlation between previous and subsequent scores.

## Results

### Characteristics of the participants

A total of 945 breast cancer patients participated in this survey, whereas 67 patients were excluded because the questionnaire was incomplete or the answer time was less than 2 min. Finally, 878 patients were included in the study, with an effective response rate of 92.91%. All patients were female, and the mean age of patients was 50.87 years (range 20– 85). Details of the sociodemographic and clinical characteristics of participants were shown in Table 1.

**Table 1:** Sociodemographic and clinical characteristics of participants (*N* = 878)

Variable	Mean ± SD or n (%)
Age (years)	50.87 ± 0.422
Ethnicity	
Han	798(90.9)
Other	80(9.1)
Education	
Elementary school or lower	92(10.5)
Middle school	271(30.9)
High school	172(19.6)
College or above	343(39.1)
Marital status	
Unmarried	55(6.3)
Married	724(82.5)
Divorced	44(5.0)
Widowed	55(6.3)
Religious beliefs	
With	89(10.1)
Without	789(89.9)
Primary caregiver	
Husband	441(50.2)
Children	263(30.0)
Parents	70(8.0)
Other	104(11.8)
Health insurance	
With	832(94.8)
Without	46(5.2)
Occupation	
Unemployed	231(26.3)
Employed	265(30.2)
Resign	57(6.5)
Retired	325(37)
Breast cancer stage	
I	115(13.1)
II	486(55.4)
III	197(22.4)
IV	80(9.1)
Time since diagnosis	
<1 year	565(64.4)
1 ~ 2 years	164(66)
2 ~ 3 years	66(7.5)
>3 years	83(9.5)
Recurrence	
Yes	145(16.5)
No	733(83.5)
Treatment received	
Surgery	314(35.8)
Radiotherapy	119(13.6)
Chemotherapy	495(56.4)
Endocrine therapy	146(16.6)
Immunotherapy	96(10.9)
Targeted therapy	87(9.9)
Neoadjuvant therapy	5(0.6)
Breast surgery performed	
None	178(20.3)
Breast conserving surgery	130(14.8)
Mastectomy	532(60.6)
Mastectomy with reconstruction	38(4.3)

SD: standard deviation

### Item analysis

As shown in Table 2, the CR values of each item ranged from 11.29 to 41.39 (> 3.0), all of which were statistically significant (*P* < 0.01); and item-total correlations for the 24 items ranged from 0.530 to 0.747, which were all statistically significant (*P* < 0.01). In addition, the deletion of any item resulted in a decrease in the overall Cronbach’s coefficient of the scale. These results indicate that the Chinese version of the SBCS items had good discrimination and all 24 items were retained.

**Table 2:** Item analysis and reliability of the Chinese version of SBCS (*N* = 878)

Factor	Item	CR	ITC	Cronbach’s alpha if item deleted	Cronbach’s alpha	Split-half reliability
Factor 1					0.884	0.863
	Q18	41.04**	0.630**	0.936		
	Q19	38.63**	0.621**	0.936		
	Q14	41.39**	0.656**	0.935		
	Q15	37.63**	0.659**	0.935		
	Q17	36.30**	0.630**	0.936		
	Q16	33.47**	0.572**	0.937		
	Q13	38.47**	0.683**	0.935		
	Q11	39.66**	0.721**	0.934		
Factor 2					0.900	0.892
	Q23	24.12**	0.695**	0.935		
	Q21	36.97**	0.747**	0.934		
	Q22	29.84**	0.718**	0.934		
	Q20	31.38**	0.701**	0.935		
	Q24	28.51**	0.711**	0.934		
Factor 3					0.837	0.761
	Q2	22.54**	0.531**	0.938		
	Q1	19.36**	0.549**	0.937		
	Q4	34.46**	0.668**	0.935		
	Q3	28.86**	0.558**	0.937		
Factor 4					0.827	0.848
	Q8	11.29**	0.530**	0.937		
	Q9	19.53**	0.684**	0.935		
	Q10	20.91**	0.701**	0.935		
	Q12	19.57**	0.668**	0.935		
Factor 5					0.779	0.781
	Q7	19.04**	0.604**	0.936		
	Q6	23.06**	0.697**	0.935		
	Q5	27.49**	0.557**	0.937		
Total					0.938	0.853

SBCS: Stressors in Breast Cancer Scale; CR: Critical ratio (t); ITC, item-total correlation

**P < 0.01

### Content validity

The results of this study showed that the I-CVI was 0.80-1.00 (> 0.78), and the S-CVI was 0.975 (> 0.9), meeting the acceptance criteria.

### Construct validity

#### Exploratory factor analysis (EFA)

**Fig. 1:**
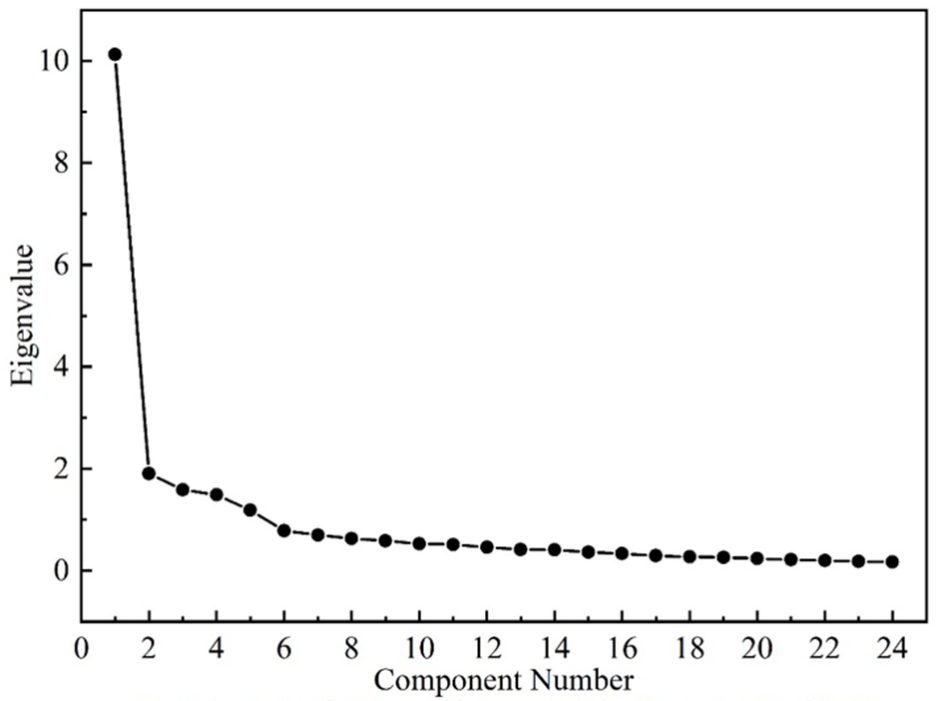
Scree plot of exploratory factor analysis for Chinese version of SBCS. SBCS: Stressors in breast cancer scale

The results of EFA showed that the Bartlett’s test of sphericity was significant (*P* < 0.001) and the KMO test was 0.917 (> 0.6), which allowed for subsequent factor analysis. PCA was used to extract the common factors by maximum variance orthogonal rotation, eventually obtaining five common factors with eigenvalues greater than 1, explaining 17.15%, 16.30%, 12.54%, 12.26%, and 9.70% of the total variation, respectively, with a cumulative variance contribution of 67.95% (Shown in Table 3). Similarly, the scree plot also showed (see Fig. 1) that after the fifth point, the fold slope gradually flattened out. Based on theoretical inferences and semantic analysis, extracted factor 1 (items 11, 13–19), factor 2 (items 20–24), factor 3 (items 1–4), factor 4 (items 8–10, 12), and factor 5 (items 5–7) were designated as interpersonal relationship and healthcare strains, worries and concerns about the future, physical appearance and sex strains, and daily difficulties and health, respectively. The factor loadings were shown in Table 3, and the factor loadings of each item on the corresponding common factor ranged from 0.557 to 0.817, all > 0.400, and no items were deleted.

**Table 3:** Exploratory factor analysis and convergent validity of the Chinese version of SBCS (*N* = 439)

Item	Factor 1	Factor 2	Factor 3	Factor 4	Factor 5	AVE	CR
Q18	**0.816**	0.217		0.177		0.638	0.934
Q19	**0.783**	0.175	0.181	0.169			
Q14	**0.672**	0.225	0.363	0.130			
Q15	**0.664**	0.163	0.103	0.118	0.406		
Q17	**0.636**	0.235		0.189	0.363		
Q16	**0.565**			0.185	0.431		
Q13	**0.557**	0.339	0.233	0.289			
Q11	**0.480**	0.347	0.274	0.436			
Q23	0.134	**0.811**	0.148	0.199	0.161	0.539	0.851
Q21	0.249	**0.770**	0.168	0.282			
Q22	0.190	**0.767**	0.242	0.282			
Q20	0.301	**0.708**	0.144		0.264		
Q24	0.345	**0.695**	0.109		0.332		
Q2	0.130		**0.817**		0.226	0.701	0.903
Q1	0.118		**0.760**		0.334		
Q4	0.156	0.378	**0.721**	0.189			
Q3	0.150	0.216	**0.712**	0.259			
Q8	0.146			**0.752**	0.320	0.541	0.825
Q9	0.183	0.293	0.165	**0.727**	0.168		
Q10	0.297	0.237	0.194	**0.711**	0.122		
Q12	0.294	0.294	0.105	**0.597**	0.185		
Q7		0.203		0.399	**0.734**	0.712	0.881
Q6	0.184	0.387	0.312	0.127	**0.640**		
Q5		0.139	0.326	0.258	**0.571**		
Eigenvalue	10.127	1.908	1.590	1.491	1.190		
variance contribution, %	17.15	16.30	12.54	12.26	9.70		
Cumulative variance contribution, %					67.95		

SBCS?Stressors in Breast Cancer Scale; The bold values were factor loadings rotated to a common factor; AVE: average variance extracted values; CR: Composite reliability

#### Confirmatory factor analysis (CFA)

**Fig. 2:**
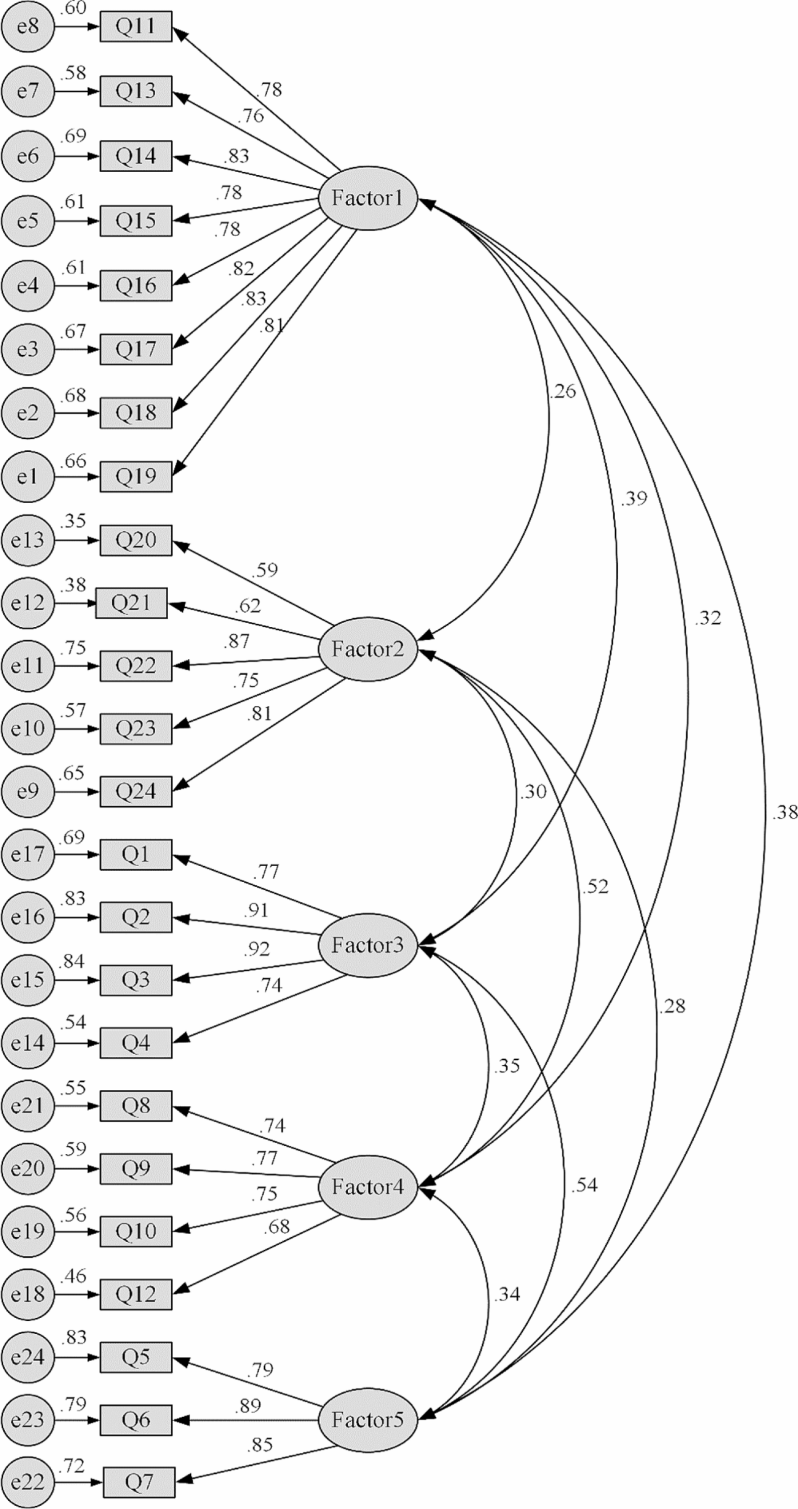
Results of confirmatory factor analysis for Chinese version of SBCS. SBCS: Stressors in Breast Cancer Scale; Factor 1: interpersonal relationship and healthcare strains; Factor 2: worries and concerns about the future; Factor 3: physical appearance and sex strains; Factor 4: daily difficulties; Factor 5: health

Generally speaking, χ2/DF values < 3 indicated a good fit and RMSEA values < 0.08 demonstrated good adaptability and good model fit [48, 49], while the remaining indicators values > 0.9 indicated a good fit; however, a value > 0.8 suggests that the model is acceptable [50]. The model fitness indices were shown in Table 4, which showed that the model had a good fit with the data. In the CFA, the five dimensions of the questionnaire were used as latent variables and the 24 items of the questionnaire were used as observed variables to plot the model, and the results of CFA are shown in Fig. 2. The factor loadings of each item ranged from 0.59 ~ 0.92.

**Table 4:** Goodness-of-fit indices of the Chinese version of SBCS (*N* = 439)

Fit indices	χ2 /DF	RMSEA	GFI	CFI	AGFI	IFI	TLI
Acceptable value	<3	<0.08	>0.8	>0.8	>0.8	>0.8	>0.8
Observed value	2.416	0.057	0.896	0.947	0.871	0.947	0.939

SBCS: Stressors in Breast Cancer Scale; χ2/DF, chi-square/degree of freedom; RMSEA, root mean square error of approximation; GFI, goodness of fit index; CFI, comparative fit index; AGFI, adjusted goodness of fit; IFI, incremental fit index; RFI, relative fit index; NFI, normed fit index

### Convergent validity and discriminant validity

As shown in Table 3, the AVE values ranged from 0.539 to 0.712 (> 0.5) and the CR values ranged from 0.825 to 0.934 (> 0.6), which showed the scale had a good convergent validity; and as shown in Table 5, the $\sqrt{AVE}$values were all greater than the correlation coefficients, which indicated that the scale had a good discriminant validity.

**Table 5:** Discriminant validity of the Chinese version of SBCS

	Factor 1	Factor 2	Factor 3	Factor 4	Factor 5
Factor 1	**0.638**				
Factor 2	0.263**	**0.539**			
Factor 3	0.390**	0.300**	**0.701**		
Factor 4	0.319**	0.521**	0.345**	**0.541**	
Factor 5	0.385**	0.282**	0.539**	0.342**	**0.712**
$\surd AVE$	0.799	0.734	0.837	0.736	0.844

SBCS: Stressors in Breast Cancer Scale; figures in bold represent AVE

**P < 0.01

### Criterion-related validity

The Chinese version of the PSS was used as the validity tool. The normality test showed that the total scores of the Chinese version of the PSS scale and the total scores of the SBCS scale were both normally distributed, so the Pearson correlation analysis was used to conduct the correlation analysis. The results showed that the total scores of the two scales were positively correlated (*r* = 0.511, *P* < 0.01).

### Reliability

As shown in Table 2, the Cronbach’s alpha coefficient of the total scale was 0.938, and the five dimensions ranged from 0.779 to 0.900; the split-half reliability (Spearman-Brown coefficient) of the total scale was 0.853, and the five dimensions ranged from 0.761 to 0.892; and the retest reliability was 0.855. These results indicated that the scale had good reliability.

## Discussion

At present, few studies on stress in breast cancer patients have been reported in China, and one of the reasons for this is the lack of native stress measurement tools for breast cancer patients. In order to scientifically and effectively assess the stress of breast cancer patients, we translated the English version of the SBCS into Chinese and comprehensively analyzed the psychometric properties of a sample of 878 women diagnosed with breast cancer, including item analysis, validity analysis, and reliability analysis. The scale was first applied to the Chinese population with good validity and reliability, which helps to identify breast cancer patients with high stress and give them targeted supportive care, thus relieving stress and improving physical and mental health.

Item analysis is one of the key steps in the scale revision process as it helps to test the quality of items. In this study, the critical ratio method, correlation coefficient method, and homogeneity test were used to examine the discriminability of the items. The results of the critical ratio method showed that the CR values were greater than 0.3, all of which were statistically different. Item-total correlations were all 0.530 and above, showing moderate to high correlation. In addition, deletion of any item resulted in a decrease in the total Cronbach’s alpha coefficient of the scale. The above indicates that the 24 items in the Chinese version of the SBCS can well reflect the stressors of breast cancer patients. Different from this study, the English version of the SBCS only analyzed the items through the correlation coefficient method, which is one of the strengths of this study.

Validity refers to the degree to which a measurement instrument or tool can accurately measure the thing to be measured [51]. This study evaluated the validity of the Chinese version of the SBCS in five aspects: content validity, construct validity, convergent validity, discriminant validity, and criterion-related validity. Five experts from different fields were invited to evaluate the content validity. The I-CVI was > 0.78, and the S-CVI was > 0.9, indicating that the scale has good content validity and is suitable for evaluating the stress of Chinese breast cancer patients [38, 39]. Construct validity reflects the degree of integration of a scale with the theoretical or conceptual framework on which it is based and is often measured by EFA [52].The English version of the SBCS demonstrates a five-factor structure: physical appearance and sex strains, health and daily difficulties, interpersonal relationship strains, healthcare strains, and worries and concerns about the future. In this study, five common factors were also extracted by EFA, but the Chinese version of the SBCS differed slightly in dimensions and item divisions from the English version. The Chinese version combined the dimensions of interpersonal relationship strains and healthcare strains into one, whereas the dimension of health and daily difficulties was divided into two. These differences may be partly due to cultural or population differences. Therefore, further research is required to explore the potential dimensions of the scale in different cultural backgrounds and populations.

The English version of SBCS’s items 10 and 12 were assigned to the dimension of interpersonal relationship strains, but were classified to the dimension of daily difficulties in this study, which may be attributed to cultural differences and different values. In Western cultures, going out with friends is seen as a social activity that helps maintain and strengthen interpersonal relationships. In contrast, in Chinese culture, going out with friends is often viewed as a leisure activity to relax and reduce stress. Similarly, caring for family members is seen as a special interpersonal relationship that requires attention and dedication in Western cultures, while in Chinese culture, it is considered a daily event that reflects love and responsibility towards family members. Therefore, further research is necessary to explore these differences. According to the EFA, the contribution of the five factors extracted in this study to the cumulative variance was 67.95%, and the factor loadings were 0.59 to 0.92, indicating that the items can well reflect the stress of breast cancer patients for recognize changes in patients’ conditions. All the fitness indices met the judgment criteria, indicating that the Chinese version of the SBCS scale has a good overall fit. In addition, AVE values were > 0.5, CR values were > 0.6, and √AVE values were greater than the correlation coefficients, further indicating that the scale was reliable and valid in different aspects. The correlation coefficient between the Chinese version of the SBCS total score and the Chinese version of the PSS total score was 0.511, with good criterion-related validity, which was consistent with the results of previous studies [23]. Overall, the Chinese version of the SBCS scale has suitable validity among Chinese breast cancer patients.

This study examined the reliability of the Chinese version of the SBCS in terms of internal consistency, split-half reliability, and test-retest reliability. The Cronbach’s alpha coefficient was 0.938, which was slightly lower than the English version’s 0.95 [23]. The split-half reliability was 0.853 and the test-retest reliability was 0.855, indicating that the scale has good internal consistency reliability and good time stability.

### Limitations

There are some limitations to this study. First, the SBCS is a self-report scale which means the results are inevitably biased. Second, the use of convenience sampling restricts the generalizability of the findings. Third, there is a lack of research on SBCS, making it difficult to make appropriate comparisons with previous reports. Fourth, we did not examine the invariance of time to diagnosis for different types of cancer, as only 9.5% of patients in our study have been diagnosed with cancer for ≥ 3 years. Lastly, this study fails to conduct applied research and has not yet explored the factors associated with stressors affecting breast cancer patients. Despite these limitations, our study provides a validated tool specifically designed for assessing stressors in Chinese breast cancer patients. This tool can be used to provide evidence or education on nursing practice to improve the quality of life and stress management for breast cancer patients.

## Conclusion

The English version of the SBCS scale has been successfully translated and culturally adapted in China, and its psychometric properties have been validated in breast cancer patients. The scale can effectively assess the stress status of Chinese breast cancer patients and identify changes in stress timely, which is essential for nursing staff to develop educational plans and interventions to alleviate stress and improve quality of life.

## Data Availability

The datasets generated and/or analyzed during the current study are not publicly available to preserve anonymity of the respondents but are available from the corresponding author on reasonable request.
